# Dieckol Reduces Muscle Atrophy by Modulating Angiotensin Type II Type 1 Receptor and NADPH Oxidase in Spontaneously Hypertensive Rats

**DOI:** 10.3390/antiox10101561

**Published:** 2021-09-30

**Authors:** Seyeon Oh, Jin Young Yang, Chul Hyun Park, Kuk Hui Son, Kyunghee Byun

**Affiliations:** 1 Functional Cellular Networks Laboratory, Lee Gil Ya Cancer and Diabetes Institute, Gachon University College of Medicine, Incheon 21999, Korea; seyeon8965@gmail.com (S.O.); roswellgirl111@gmail.com (J.Y.Y.); 2 Department of Thoracic and Cardiovascular Surgery, Gil Medical Center, Gachon University, Incheon 21565, Korea; cdgpch@gilhospital.com; 3 Department of Anatomy & Cell Biology, Gachon University College of Medicine, Incheon 21936, Korea

**Keywords:** muscle atrophy, hypertension, Th17/Treg balance, *Ecklonia cava* extract, dieckol

## Abstract

The renin–angiotensin system is involved in the development of hypertension and sarcopenia. Increased levels of angiotensin II (Ang II) lead to upregulation of Ang II type 1 receptor (AT1R), which results in increasing reactive oxygen species (ROS) by NAD(P)H oxidase (Nox). Increased ROS led to increased helper T17 (Th17) and decreased regulatory T (Treg) cells through HIF-1α. Increased Th17 secretes more IL-17, leading to increased NF-κB and muscle atrophy. We evaluated the effect of *Ecklonia cava* extracts (ECE) and dieckol (DK) on attenuating muscle atrophy by decreasing AT1R and NOX activity in spontaneous hypertensive rats (SHRs). The serum levels of Ang II and expression of AT1R in the muscle were higher in SHRs than in normotensive animals of Wistar–Kyoto rats (2.4 and 1.8 times higher than WKY, respectively). The expression of AT1R decreased by ECE or DK (0.62 and 0.84 times lower than SHR, respectively). In SHRs, the expression of Nox 1, 2, and 4 were increased (1.2–1.15 times higher than WKY) but were decreased by the administration of ECE (0.8–0.9 times lower than SHR) or DK (0.7–0.9 times lower than SHR). The Nox activity was increased in SHRs (2.3 times more than WKY) and it was decreased by ECE (0.9 times lower than SHRs) and DK (0.9 times lower than SHRs). The expression of HIF-1α, a marker of Th17 (RORγt), and cytokine secreted by Th17 (IL-17) was increased in SHRs and was decreased by ECE or DK. The marker of Treg (Foxp3) and cytokine secreted from Treg cells (IL-10) was decreased in SHRs and was increased by ECE or DK. The expression of NF-κB/IL-1β/TNF-α and MuRF-1/MAFbx/atrogin-1 was increased in SHRs and these were decreased by ECE or DK. The cross-sectional area of muscle fiber was decreased in SHRs (0.7 times lower than WKY) and was increased by ECE (1.3 times greater than SHR) or DK (1.5 times greater than SHR). In conclusion, ECE or DK leads to a decreased expression of AT1R and Nox activity which modulates Th17/Treg balance and consequently, decreased muscle atrophy.

## 1. Introduction

The renin–angiotensin system (RAS) is an essential and dominant regulatory pathway of blood pressure [1]. It is also involved in muscle atrophy or sarcopenia [2].

It has been revealed that angiotensin II (Ang II) leads to upregulation of NAD(P)H oxidase (Nox) through Ang II type 1 receptor (AT1R) [3]. Ang II increases the production of reactive oxygen species (ROS) by activating various Nox, such as Nox1, Nox2, Nox4, and Nox5, and leads to upregulation of cell adhesion molecules and increases the synthesis of proinflammatory mediators and growth factors in the vascular system [3,4]. ROS, which is increased by Ang II, is involved in hypertrophy of cardiac myocytes and vascular smooth muscle cells, dysfunction of endothelial cells, and hypertension [5,6,7]. Nox-induced ROS production is also involved in skeletal muscle atrophy [8,9]. Additionally, infusion of Ang II for 4 weeks directly caused muscle atrophy in mice [10].

The progenitor CD4+ helper T cells could differentiate into helper T1 (Th1), Th2, Th17, and regulatory T (Treg) cells [11]. Differentiation into Th17 cells is induced by signal transducer and activator of transcription 3 (STAT3) and retinoid acid-related orphan nuclear receptor t (RORt) [12]. The transcription factor forkhead box P3 (Foxp3) acts as a negative regulator of RORt and leads to the differentiation of Tregs cells [13]. Th17 cells are involved in chronic autoinflammatory diseases, such as rheumatoid arthritis, by producing cytokines such as interleukin (IL)-17A, IL-17F, IL-21, IL-22, and IL-23 [14]. Meanwhile, Treg cells show anti-inflammatory actions by secreting anti-inflammatory cytokines, such as IL-10 and transforming growth factor-beta (TGF-β) [14,15].

Both Th17 cells and Tregs are known to be involved in hypertension. Th17 cells are related to hypertension and vascular dysfunction by secretion of the cytokine IL-17 induced by Ang II [16]. Meanwhile, Treg cells attenuate endothelial dysfunction in hypertension by releasing IL-10, which decreases ROS production [17]. They are also involved in skeletal muscle repair. During muscle repair, the local expansion of Treg cells is essential and depleting anti-CD 25m Ab targeting CD4^+^CD25^hi^ Treg leads to increased muscle damage in dystrophic mice [18,19]. It is also known that Th17 cells are increased in the muscle of a denervation-induced sarcopenia model [20].

ROS is involved in facilitating differentiation to Th17 cells since Nox-deficient T cells demonstrated low ROS concentration and enhanced differentiation to nonpathogenic T cells, which produce IL-10 and TGF-β [21,22].

Under hypoxic conditions, the expression of hypoxia-inducible factor 1 alpha (HIF-1α) is increased, which is related to Th17 cell differentiation [23]. In addition, proinflammatory cytokines, such as IL-1β, IL-6, and tumor necrosis factor alpha (TNF-α), are involved in increased expression of HIF-1α [24]. HIF-1 increases differentiation to Th17 cells by direct transcriptional activation of RORγt and through the formation of a complex of RORγt and p300, which increases IL-17 [24]. Moreover, HIF-1α leads to proteasomal degradation of Foxp3 and blocks differentiation to Treg cells [24]. IL-17 upregulates nuclear factor-κB (NF-κB) by binding to the IL-17 receptor [25]. The upregulation of NF-κB results in increasing the proteolytic activity of the ubiquitin–proteasome system (UPS) by increasing the expressions of muscle atrophy F-box (MAFbx/atrogin-1) and muscle-specific RING-finger protein 1 (MuRF-1), consequently increasing muscle protein degradation [26,27,28]. NF-κB also mediates the increase in proinflammatory cytokines, such as TNF-α, IL-1, and IL-6 in various cells [29]. Furthermore, TNF-α upregulates MAFbx/atrogin-1, MuRF-1, and atrophy of C_2_C_12_ myotubes [30]. Similar to TNF-α, IL-1β leads to a decrease in the size of myotubes and upregulation of NF-κB signaling, which consequently results in increased expression of MAFbx/atrogin-1 and MuRF-1 [31].

It has been reported that *Ecklonia cava* contains various biologic molecules which shows antioxidant, anti-inflammatory, and antihypertensive effects [32,33,34,35].

Dieckol (DK), a phlorotannin in *Ecklonia cava*, has been especially noticed for its antihypertensive effects, since it involves in inhibiting angiotensin 1-converting enzyme [36]. Moreover, DK is known to attenuate hypertensive nephropathy by decreasing the serum levels of Ang II and decreasing the expression of (AT1R) in the kidney of spontaneously hypertensive rat (SHR) [37]. It is also involved in modulating blood pressure by decreasing Th17 and increasing Treg in the intestine and aorta of SHRs [38]. Although it is well known that Ang II leads to increased ROS, which might increase Th17 and Th17, consequently leading to upregulation of NF-κB, which increases muscle atrophy, it has not been fully revealed whether the changes in the balance between Th17 and Treg cells lead to muscle atrophy in SHRs, which showed an increased level of Ang II.

In this study, we evaluated whether *Ecklonia cava* extracts (ECE) and DK could modulate the balance of Th17/Treg cells by decreasing Nox activity, which had been enhanced by Ang II in SHRs. We hypothesized that ECE and DK decreased the expression of AT1R and consequently led to a decreased production of ROS by Nox. The decreased Nox activity leads to downregulation of HIF-1α, thus decreasing the differentiation to Th17, resulting in decreased NF-κB expression. The decreased NF-κB led to decreased IL-1β and TNF-α, which attenuated muscle atrophy.

## 2. Materials and Methods

### 2.1. Preparation of ECE and Isolation of DK

*Ecklonia cava* was provided by Aqua Green Technology Co., Ltd. (Jeju, Korea). For extraction, *Ecklonia cava* was washed and naturally dried for 48 h, then the leaf blades were homogenized by grinder, 50% ethanol was added, and incubated at 85 °C for 12 h. The extracts of *Ecklonia cava* were filtered, concentrated, sterilized by heating to ≥85 °C for 40–60 min, and then spray-dried. DK, one of the representative phlorotannins present in ECE, was isolated using centrifugal partition chromatography. We finally confirmed that the purity of the DK used in the study was 93.58% [39].

### 2.2. Experiment Animals

Wistar–Kyoto (WKY) rats (male, 8 weeks old, 270 ± 4 g) and SHRs (male, 8 weeks old, 280 ± 3 g) were obtained from the Animal Resource Centre (Murdoch WA, Australia) and cared at a constant temperature of approximately 23 °C, relative humidity of 50%, and a dark/light cycle of 12 h/12 h. After one week of acclimatization, the rats were then randomly divided into four groups of 5 rats per group as follows: (1) WKY/water group were orally administered with 5 mL/kg of drinking water; (2) SHR/water group were orally administered with 5 mL/kg of drinking water; (3) in SHR/ECE group, 100 mg/kg of ECE dissolved in drinking water was orally administered in an amount of 5 mL/kg; (4) in SHR/DK group, 2.5 mg/kg of DK dissolved in drinking water was orally administered in an amount of 5 mL/kg. [35,38]. All rats were orally administered the substance using an oral zonde for each rat at 9 a.m. every morning for 4 weeks. When rats were sacrificed, whole blood was collected and serum was separated using serum-separating tube (SST; BD Vacutainer, cat. 365967, Franklin Kakes, NJ, USA). All animal experiments were performed with approval according to the ethical principles of the Institutional Animal Care and Use Committee of Gachon University (approval number: LCDI-2019-0121). All experiments were repeated thrice per animal.

### 2.3. Sample Preparation

#### 2.3.1. RNA Extraction and cDNA Synthesis

According to the manufacturer’s instructions, total RNA was isolated using RNAiso Plus (Takara, cat. 9108, Kyoto, Japan). The extracted RNA pellet was washed with a high-quality wash of 70% ethanol. Then, the pellet was air-dried and dissolved in 30 µL of diethyl pyrocarbonate-treated water. The extracted RNA samples were quantified using a Nanodrop 2000 (Thermo Fisher Scientific, Waltham, MA, USA) and was synthesized using a cDNA synthesis kit (Takara, cat. 6110A) according to the manufacturer’s instructions.

#### 2.3.2. Protein Isolation

The gastrocnemius muscle protein of rats was isolated using the EzRIPA lysis kit (ATTO Corporation, cat. WSE-7420, Tokyo, Japan). First, muscle tissues were homogenized with lysis buffer containing protease and phosphatase inhibitor. Then, protein samples were briefly sonicated and centrifuged at 14,000× *g* for 20 min at 4 °C. After collecting the supernatants and transferring it to a new tube, the protein quantification was determined using a bicinchoninic acid assay kit (BCA kit; Thermo Fisher Scientific, cat. 23225, Waltham, MA, USA).

#### 2.3.3. Paraffin-Embedded Tissue Section

The gastrocnemius muscle tissues of rats were stored in 4% paraformaldehyde (Sigma-Aldrich, cat.16005, St Louis, MO, USA) in phosphate buffered saline (PBS) at 4 °C for 12 h. The fixed tissue was washed for 1 h, and a paraffin block was processed using a tissue processor (Thermo Fisher Scientific, Waltham, MA, USA). The paraffin blocks were cut into sections (7 µm thick) using a microtome (Leica, Wetzlar, Germany) and dried at 45 °C overnight. The paraffin blocks were passed through xylene and four concentrations of ethanol (100, 95, 80, and 70%) to prepare them for staining.

### 2.4. Quantitative Real-Time Polymerase Chain Reaction (qRT-PCR)

The synthesized cDNA was used for qRT-PCR. The qRT-PCR mixtures contained 5 µL SYBR Green reagent (Takara, cat. RR430A), 1 µg cDNA template in 2 µL, and a 10 pmol primer in 0.8 µL and were dispensed in each well of a 384-well multiplate and then analyzed using CFX386 Touch (Bio-Rad, Hercules, CA, USA). Validated genes are listed in Supplementary Table S1.

### 2.5. Enzyme-Linked Immunosorbent Assay (ELISA)

AngII (MybioSource, cat. MBS705139, San Diego, CA, USA) in the serum, NADP/NADPH^+^ ratio (Abcam, cat. ab65349, Waltham, MA, USA) and superoxide dismutase activity (Abcam, cat. Ab65354) in the gastrocnemius muscle of each group were determined using the appropriate kit, following the manufacturer’s instructions.

### 2.6. Immunoblot Analysis

Equal amounts (30 µg) of muscle proteins were separated on 10% polyacrylamide gels and transferred to a polyvinylidene fluoride membrane (Millipore, cat. IPVH00100, Burlington, MA, USA). The membranes were incubated with NOX1 antibodies (1:3000, Novus Biologicals, cat. NBP1-31546, Abingdon, UK), NOX2 antibodies (1:1000, Novus Biologicals, cat. NBP1-41291), NOX4 antibodies (1:500, Novus Biologicals, cat. NBP1-58849), and GAPDH (1:5000, CliniSciences, cat. CSB-MA000184, Nanterre, France) for 12 h at 4 °C and then washed three times with tris-buffered saline containing 0.1% tween 20 (TTBS). Then, the membranes were incubated using a secondary antibody (Vector Laboratories, cat. PI1000, Burlingame, CA, USA). After washing three times with TTBS, enhanced chemiluminescence (ECL; GE Healthcare, cat. GERPN2232, Chicago, IL, USA) detection reagent was used to visualize the immunoreactivity proteins on the membrane.

### 2.7. Immunohistochemistry Using 3,3-Diaminobenzidine

To block endogenous peroxidase activity, deparaffinated gastrocnemius muscle tissue slides were incubated in 0.3% hydrogen peroxide for 30 min at room temperature. The slides were rinsed three times with PBS and then incubated with HIF-1α antibodies (1:400, LSBio, cat. LS-B650, Seattle, WA, USA), IL-17A antibodies (1:50, Santa Cruz Biotechnology Inc., cat. sc-293132, Dallas, TX, USA), and IL-10 antibodies for 24 h at 4 °C in normal serum. The probed slides were washed thrice with PBS and incubated in a biotinylated secondary antibody using the ABC kit (Vector Laboratories Inc., cat. 6100) for 2 h at room temperature, and then washed three times with PBS. The tissue slides were incubated with 3,3′-diaminobenzidine (DAB; cat. D8001, Sigma-Aldrich, St Louis, MO, USA) for 15 min and washed with running water. For nuclei staining, tissue slides were immersed in hematoxylin solution for 1 min and then mounted using dibutyl phthalate polystyrene xylene (DPX; cat. 06522, Sigma-Aldrich) mounting solution. The stained tissues were photographed under an optical microscope (Olympus Optical Co., Tokyo, Japan) and analyzed using ImageJ (NIH, Bethesda, MD, USA) software.

### 2.8. Hematoxylin and Eosin Staining

Hematoxylin and eosin staining was used to determine the cross-sectional area of the muscle fiber. The muscle tissue slides were incubated with hematoxylin (DAKO, cat. CS700, Glostrup, Denmark) for 1 min, rinsed in distilled water for 10 min, and placed in eosin Y solution (Sigma-Aldrich, cat.318906) for 1 min at room temperature. Nuclei were detected by their blue appearance, and cytoplasm was detected by light-pink coloration, and the completed slides were observed under an optical microscope (Olympus Optical Co., Nagano, Japan). The mean cross-sectional area (CSA) of muscle fiber was measured using ImageJ (NIH, Bethesda, MD, USA). Histological analyses were conducted blindly, and three operators conducted at least three replicates of each analysis.

### 2.9. Statistical Analysis

Nonparametric tests were performed in this study. First, a Kruskal–Wallis test was used to determine the significance of differences among four groups. If a significant difference was confirmed using the Kruskal–Wallis test, then multiple comparisons were performed using the Mann–Whitney U-test. Results were presented as mean ± SD, and statistical significance was set as follows: *, versus WKY/water; $, versus SHR/water; and #, versus SHR/ECE. Statistical analysis was performed using SPSS v.22 (IBM Corporation, Armonk, NY, USA).

## 3. Results

### 3.1. ECE and DK Decreased Serum Ang II and the Expression of AT1R in the Muscle

**Figure 1:**
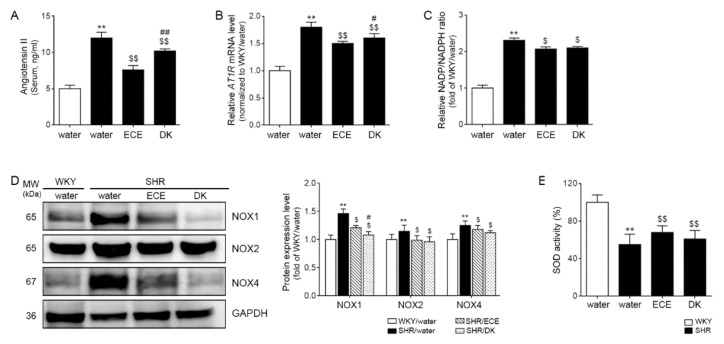
Regulatory effect of ECE and DK on serum Ang II, AT1R, and ROS production in the muscle of SHRs. (**A**) The Ang II level in serum increased following SHR/water and decreased following ECE or DK treatment; (**B**) the expression levels of *AT1R* mRNA in muscle increased following SHR/water and decreased following ECE or DK treatment. The mRNA levels were measured by qRT-PCR, normalized versus *actb*, and expressed relative to the WKY/water group levels. (**C**) The NADP^+^/NADPH ratio in muscle was increased following SHR/water and decreased following ECE or DK treatment. (**D**) The expression levels of protein in ROS production (Nox1, NoxX2, and Nox4) in muscle were increased in SHR/water groups and decreased in ECE- or DK-treated groups via immunoblotting. (**E**) The SOD activity in the muscle decreased following SHR/water and increased following ECE or DK treatment. Data are presented as mean ± SD. For each of the 4 groups. *n* = 5. **, *p* < 0.01, vs. WKY/water; $, *p* < 0.05 and $$, *p* < 0.01, vs. SHR/water; #, *p* < 0.05 and ##, *p* < 0.01, vs. SHR/ECE (Mann–Whitney U test). AT1R, angiotensin II type 1 receptor; DK, dieckol; ECE, *Ecklonia cava* extract; NADP, nicotinamide-adenin-dinucleotide phosphate; NADPH, NADP hydrogen; Nox, NADPH oxidase; SHR, spontaneously hyper-tensive rat; SOD, superoxide dismutase; WKY, Wistar Kyoto rat.

The serum level of Ang II was significantly 2.4 times higher in the SHRs than in the control animals of the WKY rats, and it was more significantly decreased by 0.62 and 0.84 times, respectively, than SHRs after administering ECE and DK. The decreasing effect was significantly more prominent in the ECE than in the DK administration group (Figure 1A).

The expression of AT1R was significantly (1.83 times) higher in SHRs than WKY and was significantly decreased by ECE and DK administration (0.83 and 0.88-fold lower than SHR, respectively). However, the decreasing effect was more prominent in ECE than in the DK administration group (Figure 1B).

### 3.2. ECE and DK Decreased NADP^+^/NADPH Levels and the Expression of Noxs and Increased Activity of SOD in the Muscle

NADP^+^/NADPH levels were measured to evaluate the activity of NADPH oxidase during ROS generation [40]. NADP^+^/NADPH level was significantly (2.31-fold) higher in the SHRs than in the WKY rats. It was significantly decreased by 0.9 and 0.91 times by ECE or DK administration; however, the decreasing effect was not significantly different between ECE and DK (Figure 1C). The expression of Nox1 was significantly (1.46 times) higher in the muscle of the SHRs than in that of the WKY rats and was significantly decreased by ECE and DK administration (0.83 and 0.74-fold lower than SHR, respectively). The decreasing effect was more prominent in the DK than in the ECE. The expression of Nox2 and Nox4 was significantly 1.15 and 1.25 times higher in the SHRs than in the WKY rats, respectively, and those were significantly decreased by ECE and DK administration (Nox2: 0.86 and 0.83 times lower than SHR, respectively; Nox4: 0.94 and 0.9 times lower than SHR, respectively). The decreasing effect was not significantly different between ECE and DK (Figure 1D).

The SOD activity of SHRs was significantly (0.55 times) lower than that of WKY rats. It was significantly increased by ECE or DK administration (1.24 and 1.11-fold higher than SHR, respectively). The increasing effect was not significantly different between ECE and DK (Figure 1E).

### 3.3. ECE and DK Decreased the Expression of HIF-1α, p300, and STAT3 in the Muscle

**Figure 2:**
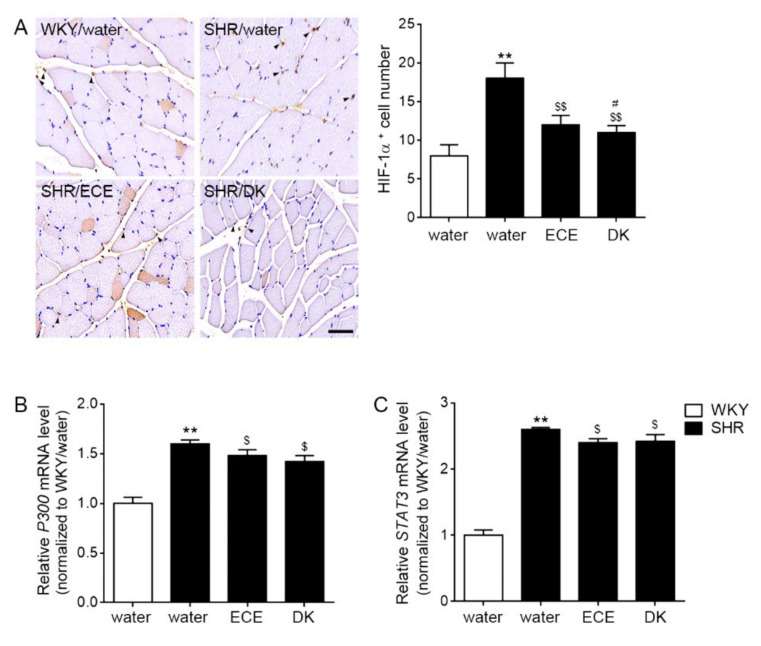
Reduction effects of ECE and DK on the expressions of HIF-1α, P300, and STAT3 in the muscle of the SHRs. (**A**) The positive cell number of HIF-1a in muscle increased following SHR/water and decreased following ECE or DK treatment by immunohistochemistry. The quantitative graph was measured signals of HIF-1α expressed in the nucleus. (**B**,**C**) The *P300* and *STAT3* mRNA expression levels in muscle increased following SHR/water and decreased following ECE or DK treatment. The mRNA levels were measured using qRT-PCR, normalized versus *actb*, and expressed relative to the WKY/water group levels. Data are presented as mean ± SD. For each of the 4 groups. *n* = 5. **, *p* < 0.01, vs. WKY/water; $, *p* < 0.05 and $$, *p* < 0.01, vs. SHR/water; #, *p* < 0.05, vs. SHR/ECE (Mann–Whitney U test). DK, dieckol; ECE, *Ecklonia cava* extract; HIF-1α, hypoxia inducible factor 1 alpha; SHR, spontaneously hyper-tensive rat; STAT3, signal transducer and activator of transcription 3; WKY, Wistar Kyoto rat.

The expression of HIF-1α was significantly (2.25 times) higher in the muscle of the SHRs than in that of the WKY rats, and those were significantly decreased 0.66 and 0.61 times than SHRs, respectively, by ECE or DK administration. The decreasing effect was more prominent in the DK than in the ECE (Figure 2A). The expression of p300 (1.63 times) and STAT3 (2.6 times) were significantly higher in the muscle of the SHRs than in that of the WKY rats, and those were significantly decreased by ECE or DK administration (p300: 0.91 and 0.87-fold lower than SHR, respectively; Stat3: 0.93 and 0.93-fold lower than SHR, respectively). The decreasing effect was not significantly different between DK and ECE (Figure 2B,C).

### 3.4. ECE and DK Decreased Th17, Increased Treg, and Decreased the Expression of NF-κB/IL-1β/TNF-α

**Figure 3:**
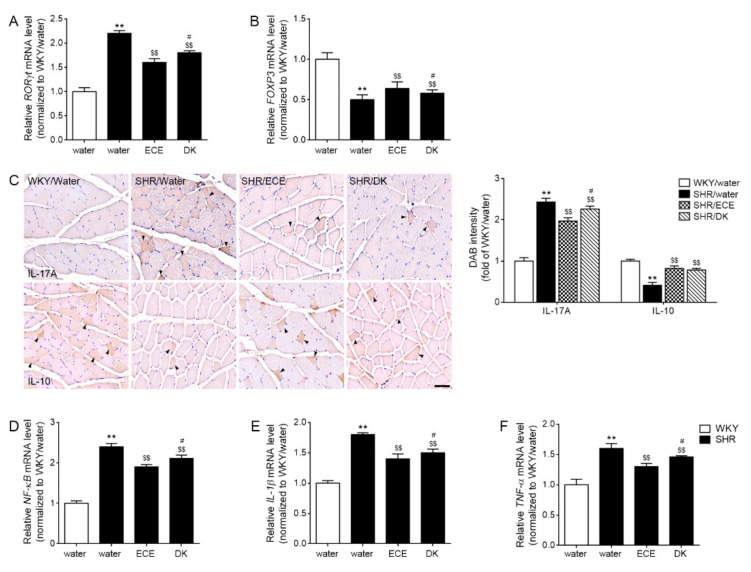
Regulatory effects of ECE and DK on decreasing factors related with Th17 differentiation and inflammatory cytokines in the muscle of SHRs. (**A**) The expression levels of RORγt in muscle were increased following SHR/water and decreased following ECE or DK treatment. (**B**) The expression levels of FOXP3 in muscle were decreased following SHR/water and increased following ECE or DK treatment. (**C**) The intensity of IL-17A in muscle was increased following SHR/water and decreased following ECE or DK treatment via immunohistochemistry. However, the intensity of IL-10 in muscle was decreased following SHR/water and increased following ECE or DK treatment. (**D**–**F**) The expression of NF-κB (**D**), IL-1β (**E**), and TNF-α (**F**) mRNA levels in muscle was increased following SHR/water and decreased following ECE or DK treatment. The mRNA levels were measured using qRT-PCR, normalized versus *actb*, and expressed relative to the WKY/water group levels. Data are presented as mean ± SD. For each of the 4 groups. *n* = 5. **, *p* < 0.01, vs. WKY/water; $$, *p* < 0.01, vs. SHR/water; #, *p* < 0.05, vs. SHR/ECE (Mann–Whitney U test). DK, dieckol; ECE, Ecklonia cava extract; FOXP3, forkhead box P3; IL-1β, interleukin-1beta; IL-10, interleukin-10; IL-17A, interleukin-17A; NF-κB, nuclear factor kappa-light-chain-enhancer of activated B cells; RORγt, retinoic-acid-receptor-related orphan nuclear receptor gamma t; SHR, spontaneously hypertensive rat; TNF-α, tumor necrosis factor-alpha; WKY, Wistar Kyoto rat.

The expression of RORγt was 2.21-fold higher in the muscle of the SHRs than in that of the WKY rats. It was decreased 0.74 and 0.82-fold by ECE and DK administration, respectively. The decreasing effect was more prominent in the ECE than in the DK (Figure 3A). The expression of FOXP3 was 0.54 times lower in the muscle of the SHRs than in that of the WKY rats. It was increased 1.19 and 1.07 times by ECE and DK administration, respectively. The increasing effect was more prominent in the ECE than in the DK (Figure 3B).

The expression of IL-17A was 2.43-fold higher in the muscle of the SHRs than in that of the WKY rats. It was decreased by ECE and DK administration (0.81 and 0.93 times lower than SHR, respectively). The decreasing effect was more prominent in the ECE than in the DK. The expression of IL-10 was 0.62 times lower in the muscle of the SHRs than in that of the WKY rats. It was increased 1.15 and 1.08 times by ECE and DK administration. The increasing effect was not significantly different between ECE and DK (Figure 3C).

The expressions of NF-κB, IL-1β, and TNF-α were 2.42, 1.83, and 1.63 times higher in the muscle of the SHRs than in that of the WKY rats, respectively. These expressions were decreased by ECE and DK administration (NF-κB: 0.79 and 0.87 times lower than SHR, respectively; IL-1β: 0.77 and 0.84 times lower than SHR, respectively; TNF-α: 0.81 and 0.9 times lower than SHR, respectively). The decreasing effect was more prominent in the ECE than in the DK (Figure 3D–F).

### 3.5. ECE and DK Decreased the Expression of MURF-1 and MAFbx/atrogin-1 and Increased the Cross-Sectional Area of Muscle Fibers

**Figure 4:**
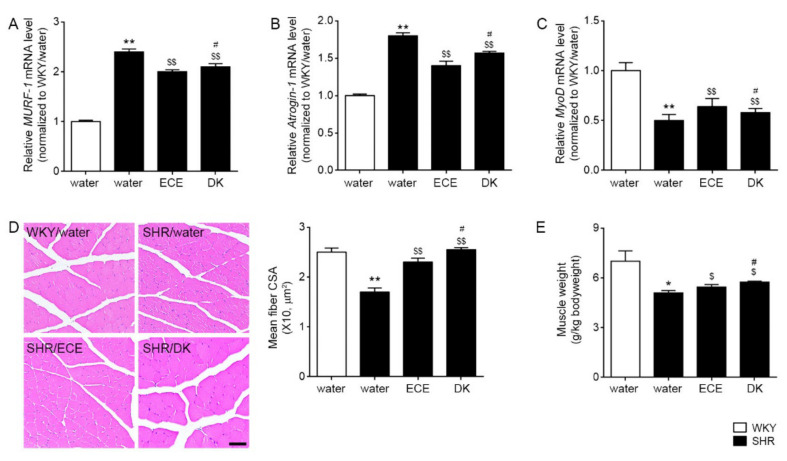
Regulatory effects of ECE and DK on muscle degradation and muscle atrophy of SHRs. (**A**,**B**) The expression levels of MURF-1 and MAFbx/atrogin-1 in muscle were increased following SHR/water and decreased following ECE or DK treatment. (**C**) The expression levels of MyoD in muscle decreased following SHR/water and increased following ECE or DK treatment. The mRNA levels were measured by qRT-PCR, normalized versus *actb*, and expressed relative to the WKY/water group levels. (**D**) The cross -sectional area of the muscle fiber was decreased following SHR/water and increased following ECE or DK treatment via hematoxylin and eosin staining. (**E**) The muscle weight/body weight was decreased by SHR/water and increased by administrating ECE or DK. Data are presented as mean ± SD. For each of the 4 groups. *n* = 5. *, *p* < 0.05 and **, *p* < 0.01, vs. WKY/water; $, *p* < 0.05, and $$, *p* < 0.01, vs. SHR/water; #, *p* < 0.05, vs. SHR/ECE (Mann–Whitney U test). CSA, corss sectional area; DK, dieckol; ECE, Ecklonia cava extract; MURF-1, muscle-specific RING-finger protein 1; MyoD, myoblast determination protein 1; SHR, spontaneously hypertensive rat; WKY, Wistar Kyoto rat.

The expression of MURF-1 and MAFbx/atrogin-1 was significantly 2.43 and 1.82-folds higher in the SHRs than the WKY, respectively, and those were significantly decreased by ECE and DK administration (MURF-1: 0.91 and 0.86-folds lower than SHR, respectively; MAFbx/atrogin-1: 0.77 and 0.86-folds lower than SHR, respectively; Figure 4A,B). The expression of myoblast determination protein 1 (MyoD) was significantly 0.52-folds lower in the SHRs than WKY, and it was significantly increased 1.34 and 1.48 times by ECE or DK administration, respectively (Figure 4C). The mean cross-sectional area of muscle fibers was significantly 0.69 times lower in the SHRs than in the WKY rats and was significantly increased 1.34 and 1.48 times, respectively, by ECE or DK administration (Figure 4D). The increasing effect was higher in the DK than in the ECE. The muscle weight of gastrocnemius which divided by body weight was significantly 0.73-fold lower in the SHRs than WKY. It was significantly increased 1.07 and 1.13-fold by ECE or DK, respectively (Figure 4E). The increasing effect was more prominent in the DK in the ECE.

## 4. Discussion

Sarcopenia is defined by the loss of muscle mass and strength and decreased physical activity [41]; thus, sarcopenia leads to poor outcomes and decreased quality of life [42,43,44]. The muscle mass decreases by aging, and decreasing is accelerated by physical inactivity, malnutrition, and chronic diseases such as degenerative neurological disorders, malignancies, and cardiovascular diseases.

The prevalence of sarcopenia is higher in heart failure patients than in age-matched general populations [45].

Sarcopenia is more frequently presented in hypertensive patients than in normotensive adults (32.2 vs. 7.8%) [2]. Additionally, hypertension independently increases the risk of sarcopenia more than 6.5 times more than normal blood pressure [2]. Many studies have shown that RAS is possibly the main commonality between the pathophysiology of sarcopenia and hypertension.

Ang II induces an increased expression of Nox and ROS generation [3], and consequently, increased ROS involves developing both hypertension and sarcopenia [5,6,7,8,9]. In addition, increased ROS increases differentiation to Th17 and decreases the differentiation to Treg cells.

Increased mitochondrial ROS production is related to increased differentiation of inflammatory Th17 cells in vitro and in vivo of high glucose conditions [46]. Nox deletion in T cells demonstrated enhanced differentiation to nonpathogenic Th17 cells, secreting IL-10 [21,22]. Increasing inflammatory Th17 cells and decreasing Treg cells were known to be related to hypertension and sarcopenia [20,47].

Here, we evaluated whether ECE and DK could attenuate muscle atrophy in hypertensive animal models of SHRs by decreasing the AT1R signal pathway and Nox activity. Our study results showed that an increased serum level of Ang II in SHRs was significantly decreased by ECE or DK administration. The expression of AT1R also increased in the SHRs, but it was significantly decreased by ECE or DK administration (Figure 1A,B). The Nox activity evaluated using NADP^+^/NADPH significantly increased in the SHRs in comparison with that in normotensive animals, and it was decreased by ECE or DK administration. The expression of NOX1, NOX2, and Nox4 was significantly increased in the SHRs in comparison with that in normotensive animals, but it was significantly decreased by ECE or DK administration (Figure 1C,D).

SOD enzymes are an essential defense mechanism against superoxide radicals and catalyze the dismutation of superoxide radicals into H_2_O_2_ and O_2_ [48].

Our study showed that SOD activity of SHRs was significantly decreased and partially restored by ECE or DK administration (Figure 1E). It seemed that ECE and DK decreased ROS production induced by Ang II through decreasing Nox and increasing SOD. The decreasing Nox activity and SOD activity were accompanied with decreasing HIF-1α in our study. Expression of HIF-1α was increased in the SHRs, and it was decreased by ECE or DK administration (Figure 2A).

Th17 cell differentiation is induced by the STAT3 signaling pathway, which is regulated by IL-6 and eventually increases the transcriptional regulator RORγt [49]. Additionally, increased HIF-1α by increasing ROS production or hypoxic conditions activates RORγt directly [24]. Furthermore, HIF-1α cooperates with RORγt and p300 to activate the IL-17A gene and increase Th17 development [24]. HIF-1α leads to Foxp3 degradation, thus decreasing Treg cell differentiation [24]. HIF-1α-deficient mice showed increased Treg cells [24].

These reports are evidence that HIF-1α could modulate Th17/Treg cell balance. In our study, the expression of HIF-1α in the muscle of SHRs was significantly increased compared with that in normotensive animals. The expressions of P300 and STAT3 were also increased in the SHRs. However, these expressions were significantly decreased by ECE and DK directly (Figure 2B,C). Here, we evaluated Th17/Treg cell balance with the expressions of RORγt and Foxp3. The marker of Th17, RORγt, was increased in the SHR; however, Foxp3, a marker of Treg cells, decreased in the SHRs. The expression of RORγt was decreased by ECE or DK administration. Meanwhile, the expression of FOXP3 was increased by ECE or DK administration (Figure 3A,B). The cytokine secreting from Th17, IL-17A, was increased in the muscle of SHR. The IL-10, which was secreted from Treg cells was decreased in the muscle of SHR. The expression of IL-17A was decreased by ECE or DK administration. Furthermore, ECE or DK administration increased the expression of IL-10; however, it seemed to have modulated Th17/Treg balance (Figure 3C).

The skewed balance between Th17/Tregs to Th17 dominant condition led to increased secretion of IL-17, which upregulates NF-κB [25]. Consequently, NF-κB leads to muscle atrophy by directly upregulating UPS, such as MuRF-1 and MAFbx/atrogin-1 [26,27,28], and increasing the secretion of cytokines, such as TNF-α, IL-1, and IL-6, which eventually lead to muscle atrophy [29].

In our study, the expressions of NF-κB, IL-1β, and TNF-α were increased in the muscle of the SHRs, and these expressions were decreased by ECE or DK administration (Figure 3C–F).

Additionally, the expressions of MURF-1 and MAFbx/atrogin-1 were increased in the SHRs but were decreased by ECE or DK (Figure 4A,B).

The myogenic regulatory factors are a group of muscle-specific transcription factors, such as myogenic factor 5 (Myf5), MyoD, myogenin, and myogenic regulatory factor4 (MRF4), which are involved in skeletal muscle growth and development [50,51]. For example, it is known that the expression of MyoD is decreased in age-related sarcopenia [52].

In our study, the expression of MyoD was decreased in the SHRs, and it was increased by ECE or DK administration (Figure 4C). Moreover, the mean cross-sectional area of muscle fibers and muscle weight/body weight ratio were decreased in the SHRs, and it was restored via ECE or DK administration (Figure 4D,E).

Due to the increasing old age population, the prevalence of hypertension and sarcopenia is rapidly increasing. Hypertension is the most common cardiovascular disease, and its overall prevalence is 45%, and the prevalence in over 60 years old reaches more than 60% [53,54]. The prevalence of sarcopenia in those over 60 years old is approximately 10% [55].

Hypertension and sarcopenia have a common pathophysiology of increased Ang II and ROS production; hence, controlling Ang II is a proper approach to managing sarcopenia in hypertensive patients. In our study, the DK, which has been reported to show antihypertensive effects, also attenuated muscle atrophy in the SHRs. Both ECE and DK decreased the expression of AT1R and the activity of Nox and consequently decreased HIF-1α. Decreased HIF-1αlead to decreasing NF-κB and proinflammatory cytokines related to muscle atrophy.

## 5. Conclusions

In conclusion, ECE or DK leads to a decreased expression of AT1R and Nox activity which mod-ulates Th17/Treg balance and consequently, decreased muscle atrophy.

## Data Availability

All data are contained within the article.

## Supplementary Materials

The following are available online at https://www.mdpi.com/article/10.3390/antiox10101561/s1, Table S1: List of primers for qRT-PCR.
