# Supplementary material for: Dieckol Reduces Muscle Atrophy by Modulating Angiotensin Type II Type 1 Receptor and NADPH Oxidase in Spontaneously Hypertensive Rats
Source: Antioxidants (Basel). 2021 Sep 30;10(10):1561. doi: 10.3390/antiox10101561 (PMC8533257; doi:10.3390/antiox10101561)
Supplement: Supplementary file 1 [file antioxidants-10-01561-s001.zip › antioxidants-1359977-supplementary.pdf]

Dieckol reduces muscle atrophy by modulating angiotensin type II type 1 receptor and NADPH oxidase in spontaneously hypertensive rats

Seyeon Oh [1], Jin Young Yang [1], Chul Hyun Park [2], Kuk Hui Son [2,*] and Kyunghee Byun [1,3,*]

[1] Functional Cellular Networks Laboratory, College of Medicine, Department of Medicine, Graduate School and Lee Gil Ya Cancer and Diabetes Institute, Gachon University, Incheon 21999, Republic of Korea; seyeon8965@gmail.com (S.O.); roswellgirl111@gmail.com (J.Y.Y.)

[2] Department of Thoracic and Cardiovascular Surgery, Gachon University Gil Medical Center, Gachon University, Incheon 21565, Republic of Korea; cdgpch@gilhospital.com (C.H.P.)

[3] Department of Anatomy & Cell Biology, Gachon University College of Medicine, Incheon 21936, Republic of Korea

* Correspondence: dr632@gilhospital.com; Tel.: +82-32-460-3666, khbyun1@gachon.ac.kr; Tel.: +82-32-899-6511

Supplementary Tables

Table S1. List of primer for qRT-PCR used in this study.

Gene		Primers
actb	Forward	5′- CCG TAA AGA CCT CTA TGC CAA C -3′
	Reverse	5′- GGC AGT AAT CTC CTT CTG CAT C -3′
AT1R	Forward	5′- CAT GAT CCC TAC CCT CTA CAG C -3′
	Reverse	5′- TAA ATG ACA ATC ACC ACC AAG C -3′
P300	Forward	5′- TGG TCC TCT ACC TGA TCC ATC T -3′
	Reverse	5′- CAT CTG TCC AAA CTG GTT CAA A -3′
STAT3	Forward	5′- AAA GTC AGG TTG CTG GTC AAA T -3′
	Reverse	5′- CCA GAG TCC TTA TCA ATG CAC A -3′
RORγt	Forward	5'- AGA AGG ACT CCT ATG TGG GTG A -3′
	Reverse	5'- TGG TAA CAA TGC CAT GTT CAA T -3′
FOXP3	Forward	5'- AGT TCC TTC CCA GAG TTC TTC C -3′
	Reverse	5' - GAT AAG GGT GGC ATA GGT GAA A -3′
NF-κB	Forward	5'- GCA GTT TGA TGC TGA TGA AGA C -3′
	Reverse	5'- ACT CTG AGT TGT CCA CAG ATG C -3′
IL-1β	Forward	5′- AGT GTG GAT CCC AAA CAA TAC C -3′
	Reverse	5′- TGT GCA GAC TCA AAC TCC ACT T -3′
TNF-α	Forward	5′- CAT GGA TCT CAA AGA CAA CCA A -3′
	Reverse	5′- CCT TGA AGA GAA CCT GGG AGT A -3′
MURF-1	Forward	5′- TCG ACA TCT ACA AGC AGG AAT G -3′
	Reverse	5′- GAT TTT CTC GTC TTC GTG TTC C -3′
Atrogin-1	Forward	5′- TGA AAG TTC TTG AAG ACC AGC A -3′
	Reverse	5′- GCG TGC ATA AGG ATG TGT AGA G -3′
MyoD	Forward	5′- CTC TGA TGG CAT GAT GGA TTA C -3′
	Reverse	5′- GCT GGA CGC CTC ACT GTA GTA -3′
